# Integrative proteo-transcriptomic characterization of advanced fibrosis in chronic liver disease across etiologies

**DOI:** 10.1016/j.xcrm.2025.101935

**Published:** 2025-01-30

**Authors:** Hong Yang, Dila Atak, Meng Yuan, Mengzhen Li, Ozlem Altay, Elif Demirtas, Ibrahim Batuhan Peltek, Burge Ulukan, Buket Yigit, Tarik Sipahioglu, María Bueno Álvez, Lingqi Meng, Bayram Yüksel, Hasan Turkez, Hale Kirimlioglu, Burcu Saka, Cihan Yurdaydin, Murat Akyildiz, Murat Dayangac, Mathias Uhlen, Jan Boren, Cheng Zhang, Adil Mardinoglu, Mujdat Zeybel

**Affiliations:** 1 Science for Life Laboratory, KTH - Royal Institute of Technology, Stockholm, Sweden; 2 Department of Gastroenterology and Hepatology, School of Medicine, Koç University, İstanbul 34010, Turkiye; 3 School of Medicine, Koç University, Istanbul 34450, Turkiye; 4 SZAOMICS Biotechnology R&D, İstanbul 34010, Turkiye; 5 Department of Medical Biology, Faculty of Medicine, Atatürk University, Erzurum 25240, Turkiye; 6 Department of Pathology, School of Medicine, Acibadem Mehmet Ali Aydinlar University Istanbul 34752, Turkiye; 7 Department of Pathology, School of Medicine, Koç University, Istanbul 34010, Turkiye; 8 Department of Molecular and Clinical Medicine, University of Gothenburg and Sahlgrenska University Hospital, Gothenburg, Sweden; 9 Department of General Surgery, International School of Medicine, Medipol University, Istanbul 34010, Turkiye; 10 Centre for Host-Microbiome Interactions, Faculty of Dentistry, Oral & Craniofacial Sciences, King’s College London, London SE1 9RT, UK; 11 Clinical Trials Unit, Koç University Hospital, Istanbul 34010, Turkiye

**Keywords:** chronic liver disease, liver fibrosis, multi-omics, systems biology, non-invasive

## Abstract

Chronic hepatic injury and inflammation from various causes can lead to fibrosis and cirrhosis, potentially predisposing to hepatocellular carcinoma. The molecular mechanisms underlying fibrosis and its progression remain incompletely understood. Using a proteo-transcriptomics approach, we analyze liver and plasma samples from 330 individuals, including 40 healthy individuals and 290 patients with histologically characterized fibrosis due to chronic viral infection, alcohol consumption, or metabolic dysfunction-associated steatotic liver disease. Our findings reveal dysregulated pathways related to extracellular matrix, immune response, inflammation, and metabolism in advanced fibrosis. We also identify 132 circulating proteins associated with advanced fibrosis, with neurofascin and growth differentiation factor 15 demonstrating superior predictive performance for advanced fibrosis(area under the receiver operating characteristic curve [AUROC] 0.89 [95% confidence interval (CI) 0.81–0.97]) compared to the fibrosis-4 model (AUROC 0.85 [95% CI 0.78–0.93]). These findings provide insights into fibrosis pathogenesis and highlight the potential for more accurate non-invasive diagnosis.

## Highlights

• Characterize hepatic transcriptional profiles linked to advanced liver fibrosis
• Identify 132 circulating proteomic signatures via co-analysis with liver transcriptomics
• Use ML to propose accurate and non-invasive predictors of advanced liver fibrosis

## Graphical abstract

**Figure:**
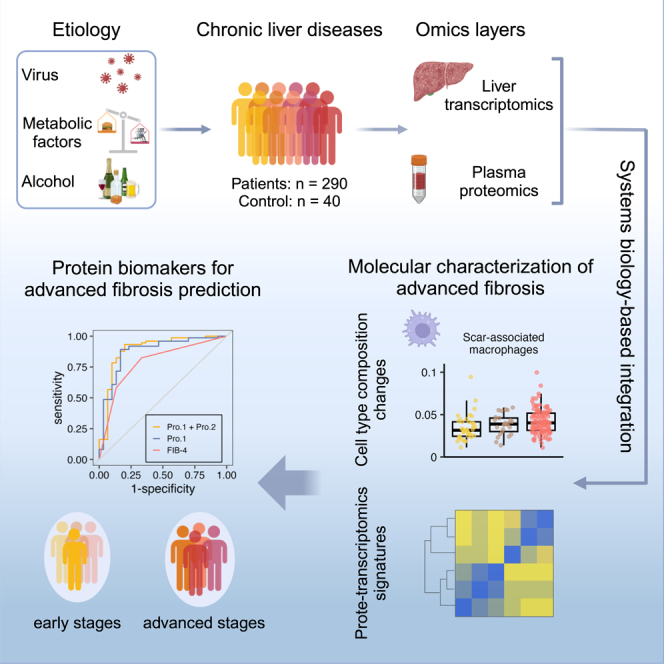

## Teaser

Advanced fibrosis in chronic liver disease is an increased risk of liver cancer. Yang et al. present an integrative proteo-transcriptomic analysis of liver and plasma samples, identifying dysregulated pathways and biomarkers such as NFASC and GDF15. Their findings reveal molecular insights and potential non-invasive diagnostic strategies for progressive fibrosis.

## Introduction

Chronic liver disease (CLD) is a significant health and economic burden globally, affecting approximately 1.5 billion people worldwide.^1^^,^^2^^,^^3^ The primary causes of CLD include chronic viral hepatitis (CVH), alcohol-related liver disease (ARLD), and metabolic dysfunction-associated steatotic liver disease (MASLD).^4^ CLD is characterized by chronic hepatic injury and persistent inflammation, leading to liver fibrosis, which may progress to cirrhosis and increase the risk of developing hepatocellular carcinoma (HCC).^5^ The severity of liver fibrosis has emerged as a critical indicator of long-term morbidity and mortality.^6^ Recent data indicate that liver fibrosis and related morbidities have become increasingly common, driven by rising rates of alcohol consumption and metabolic disorders, resulting in approximately 2 million deaths annually worldwide.^2^^,^^6^^,^^7^ However, the diagnosis of fibrosis is typically made at advanced stages, when clinical decompensation or HCC develops, due to the condition’s slow and often asymptomatic progression,^8^ a limited understanding of the underlying molecular mechanisms, and a lack of effective biomarkers for identifying patients at high risk of severe fibrosis.^9^

Liver fibrosis is a dynamic process characterized by the excessive accumulation of extracellular matrix (ECM) proteins.^10^ While technological advancements have improved our understanding of fibrogenesis,^5^^,^^11^ identifying advanced fibrosis in the general population remains challenging due to clinical heterogeneity influenced by underlying causes, comorbidities, and lifestyle factors. A systems-level characterization of metabolic and signaling dysregulation could effectively capture the signatures driving liver fibrosis caused by different etiologies. Early diagnosis of individuals at risk for progressive fibrosis could facilitate interventions such as lifestyle changes or therapeutic measures to prevent the progression to severe stages of liver disease.^12^^,^^13^^,^^14^ Despite the development of several non-invasive diagnostic methods, including serum biomarker algorithms and imaging tools to assess tissue stiffness, their variability and limited accuracy restrict their widespread use in population-level screening.^15^^,^^16^^,^^17^

Systems biology approaches, including machine learning and biological network analyses, have demonstrated potential in elucidating the underlying molecular mechanisms of complex diseases by integrating various layers of omics data.^18^^,^^19^^,^^20^ These methodologies facilitate the integration and analysis of complex biological data from genomics, transcriptomics, metabolomics, and proteomics, providing a comprehensive view of the metabolic and biological pathways involved in fibrosis and its associated cancer development. In this study, we utilized a data-driven multi-omics approach, encompassing liver transcriptomics and plasma proteomics, to thoroughly characterize patients across the pathological spectrum, from early-stage fibrosis to cirrhosis and associated HCC. This involved separate and integrative analyses of both hepatic and peripheral blood specimens, tailored to both fibrosis stage and etiology. Moreover, we integrated liver transcriptomes with human liver cirrhosis single-cell data^11^ to examine cellular composition dynamics depending on fibrosis stages and inflammation levels. Furthermore, we investigated the potential of the plasma proteome as a clinical diagnostic tool for understanding liver biology, particularly fibrosis, by assessing its predictive capabilities relative to established clinical markers. Finally, we validated these plasma-based protein signatures in an independent cohort, reinforcing their potential utility in clinical settings.

## Results

### Characteristics of cohorts, data collection, and study design

**Figure 1:**
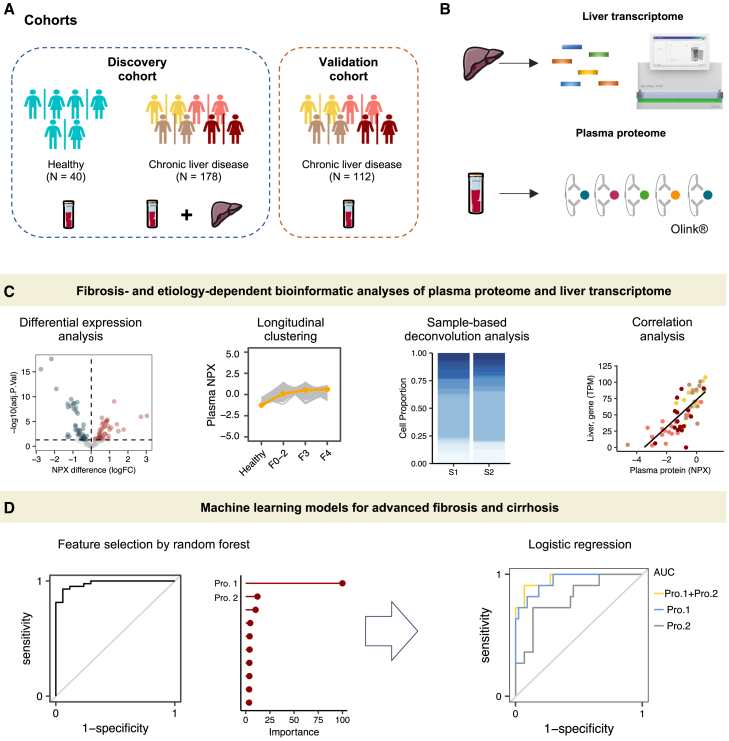
Study overview (A) Clinical cohorts. (B) Liver transcriptome sequencing was carried out on liver tissue from 178 patients with CLD (*n* = 144) or CLD and HCC (*n* = 34). Proximity extension assay-based proteomics technology was used to profile plasma samples from 330 subjects in the studied cohorts, totaling 1,463 proteins quantified. (C) Schematic representation of the bioinformatics workflow of this study, including statistical, functional, correlation, and single-cell deconvolution analyses on omics profiles. (D) Machine-learning-based classification models were used to identify potential biomarkers for advanced fibrosis and cirrhosis. Abbreviations: NPX, normalized protein expression; CLD, chronic liver disease; HCC, hepatocellular carcinoma; logFC, log fold change; AUC, area under the curve; Pro., protein; F, fibrosis; F0–2, fibrosis stage 0/1/2; S, sample.

**Figure 2:**
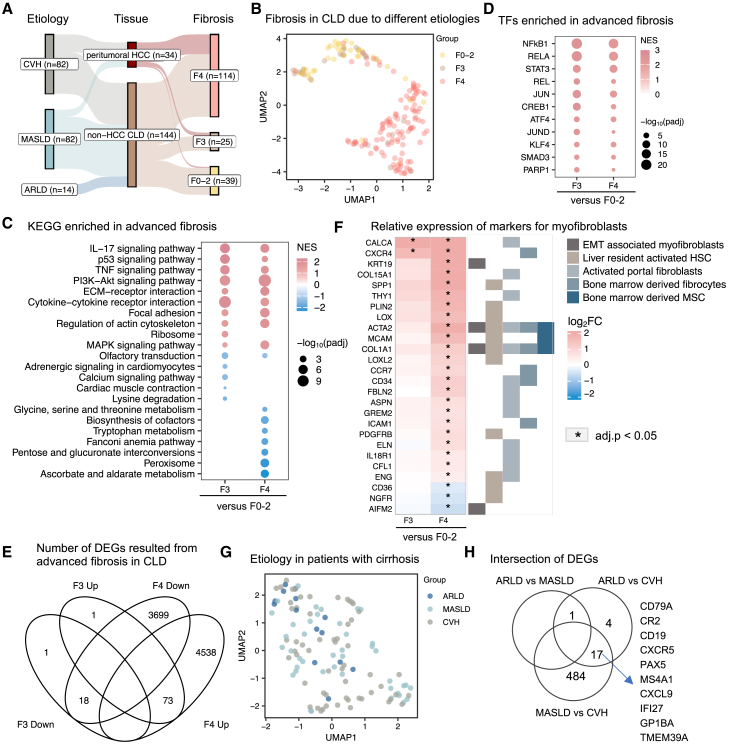
Transcriptomic signature differentiates advanced fibrosis (A) Flow diagram illustrating the number of liver tissue samples from either CLD or peritumoral CLD, categorized by each etiology and the respective histologically assessed stages of fibrosis. (B) A UMAP analysis was performed on transcriptomics data from hepatic tissues (F0–2, *n* = 39; F3, *n* = 25; F4, *n* = 114). Each data point represents a sample in the respective colored group. (C) Dot-heatmap showing the top significantly regulated Kyoto Encyclopedia of Genes and Genomes (KEGG) pathways in hepatic tissue with advanced fibrosis compared to those in F0/1/2 stages (Benjamini and Hochberg false discovery rate adjusted *p* value < 0.05), see the full list in Data S2. (D) Dot-heatmap showing the top transcription factors significantly enriched by target gene sets changed in advanced fibrotic tissue as compared to those in F0/1/2 stages (Benjamini and Hochberg false discovery rate adjusted *p* value < 0.05), see the full list in Data S2. (E) Venn diagram showing the number of differentially expressed genes (DEGs) (Benjamini and Hochberg false discovery rate adjusted *p* values < 0.05) in advanced fibrotic tissue as compared to those in the F0/1/2 stages. (F) Heatmap showing the relative expression of fibrosis marker genes in hepatic tissues with advanced fibrosis compared to those in F0/1/2 stages. (G) A UMAP analysis was performed on transcriptomics data from cirrhotic tissues resulting from different etiologies, including ARLD (*n* = 14), MASLD (*n* = 42), and CVH (*n* = 58). (H) The number of differentially expressed genes shared among pairwise comparisons across three etiologies. Abbreviations: UMAP, uniform manifold approximation and projection; HCC, hepatocellular carcinoma; CLD, chronic liver disease; CVH, chronic viral hepatitis; MASLD, metabolic dysfunction-associated liver disease; ARLD, alcohol-related liver disease; EMT, epithelial-to-mesenchymal transition; MSCs, mesenchymal stem cells; HSCs, hepatic stellate cells.

This multicenter study included two cohorts: a discovery cohort and a validation cohort, comprising a total of 330 adult participants (Table 1; Figures 1 and 2A). The discovery cohort consisted of 40 healthy individuals, 144 patients with CLD, and 34 patients with CLD and HCC (median age 53 years, interquartile range [IQR]: 42–61; 36.2% female). Among the patients with CLD, the diagnoses include CVH (40.3%), ARLD (9.7%), and MASLD (50%). For the patients with HCC, 70.6% have CVH-associated HCC, and 29.4% have MASLD-associated HCC (Table 1). The validation cohort was composed of an additional 68 patients with CLD and 44 patients with CLD-associated HCC, matched by age and gender (median age 55 years, IQR: 46–64; 24.1% female). Detailed baseline characterization of the studied cohorts regarding age, gender, clinical parameters, and diagnosis is provided in Tables 1 and S1.

**Table 1:** Baseline participant characteristics in the study

Characteristics	Discovery cohort (*n* = 218)							Validation cohort (*n* = 112)				
	Total	Total patients	MASLD	ARLD^a^	CVH	HCC	Healthy^a^	Total patients	MASLD	ARLD	CVH	HCC
	(*n* = 218)	(*n* = 178)	(*n* = 72)	(*n* = 14)	(*n* = 58)	(*n* = 34)	(*n* = 40)	(*n* = 112)	(*n* = 53)	(*n* = 1)	(*n* = 14)	(*n* = 44)
Male (*n* [%])	139 (63.8)^∗^	121 (68.1)	38 (52.8)	13 (92.9)	39 (67.2)	30 (88.2)	18 (45.0)	85 (75.9)	36 (67.9)	1	9 (64.3)	41 (93.1)
Female (*n* [%])	79 (36.2)	57 (32.0)	33 (47.2)	1 (7.1)	19 (32.8)	4 (11.8)	22 (55.0)	27 (24.1)	17 (32.1)	0	5 (35.7)	5 (6.9)
Age (years)	53 (42, 61)	56 (47, 62)	56 (47, 64)^∗^	55 ± 10	51 ± 12	60 ± 7	38 (26, 43)	55 (46, 64)	48 ± 15	62	49 ± 7	61 ± 10
BMI (kg/m^2^)	27 (24, 30)	27 (25, 31)	29 (26, 33)	28 ± 3	26 (24, 29)	28 ± 4	24 ± 2	28 (25, 31)	28 (26, 33)	29	25.7 (24, 28)	28 ± 4
Smoking (*n* [%])	31 (19.3)^∗^	26 (31.91)^∗∗∗^	11 (18.9)	5 (45.5)	8 (21.6)	2 (13.3)	5 (12.5)	33 (32.4)	10 (20.0)	1	5 (38.5)	17 (44.7)
Diabetes (*n* [%])	66 (30.4)^∗^	66 (37.2)	39 (54.1)	6 (42.9)	10 (17.2)	11 (33.3)	0	53 (48.6)	32 (60.3)	0	1 (7.7)	19 (45.2)

**Laboratory parameters**												

Albumin (g/dL)	40 (31, 46)^∗^	37 (29, 44)^∗∗∗^	42 (30, 47)^∗∗∗^	31 ± 5	35 (28, 41)^∗^	37 ± 6	48 ± 2	42 (35, 48)	47 (43, 49)	36	41 ± 4	35 ± 7
ALT (U/L)	30 (19, 51)^∗∗∗^	35 (21, 67)^∗∗^	31 (21, 57)^∗∗^	22 ± 13	41 (22, 89)	36 (22, 69)	22 ± 9	47 (30, 72)	47 (33, 68)	23	81 ± 49	36 (27, 75)
AST (U/L)	36 (24, 56)^∗∗^	39 (28, 63)	35 (25, 50)	39 ± 19	50 (35, 88)	41 (29, 79)	20 (16, 24)	46 (30, 73)	35 (28, 51)	39	72 ± 26	55 (36, 85)
Platelets (∗10^9^/L)	130 (69, 236)	110 (62, 118)^∗∗∗^	128 (65, 267)^∗∗^	81 (57, 137)	116 (59, 155)	89 (62, 119)	246 ± 53	147 (94, 230)	208 (153, 277)	128	125 (96, 229)	97 (61, 141)

**Fibrosis parameters**												

FIB-4 Index	3.1 (1.0, 6.9)	3.9 (1.6, 7.6)^∗∗∗^	2.2 (1.0, 6.4)^∗∗^	4.2 (2.9, 9.2)	4.1 (2.0, 8.0)	5.6 (3.9, 7.5)	0.6 (0.5, 0.9)	2.5 (0.9, 5.6)	1.1 (0.68, 2.3)	3,9	3.3 ± 1.6	6.0 (3.1, 12)

**Fibrosis stage (*n* [%])**												

F0	10 (5.6)	10 (5.6)	6 (8.3)	0	3 (5.2)	1 (2.9)	–	4 (3.6)	4 (7.5)	0	0	0
F1	19 (10.7)	19 (10.7)	12 (16.7)	0	6 (10.3)	1 (2.9)	–	14 (12.5)	10 (18.9)	0	2 (14.3)	2 (4.5)
F2	10 (5.7)	10 (5.7)	7 (9.7)	0	3 (5.2)	0	–	13 (11.6)	8 (15.1)	0	3 (21.4)	2 (4.5)
F3	25 (11.5)	25 (11.5)	12 (16.7)	0	8 (13.8)	5 (14.7)	–	23 (20.5)	16 (30.2)	0	5 (35.7)	2 (4.5)
F4	114 (52.3)	114 (52.3)	35 (48.6)	14 (100)	38 (65.5)	27 (79.4)	–	58 (51.8)	15 (28.3)	1 (100)	4 (28.6)	38 (86.4)

Categorical variables are presented as “frequencies (percentages).” Normally distributed variables were summarized with “mean ± standard deviation” and non-normally distributed variables were shown as “medians (interquartile ranges).”

Statistical significance between cohorts of each subgroup determined by Student’s t test for non-categorical variables and Fisher’s exact test for categorical variables, with *p* value annotations: *p* < 0.05 (∗), 0.01(∗∗), and 0.001(∗∗∗), all annotated on the discovery cohort values.

a Statistical test is not applicable since the validation cohort’s sample size (*n*) is insufficient.

The fibrosis stage in the peritumoral liver tissue of patients with HCC and liver tissue of patients with CLD was histologically evaluated using the Kleiner et al.^21^ system for MASLD and the meta-analysis of histological data in viral hepatitis (METAVIR) scoring systems for CVH. We performed transcriptomics profiling on 178 liver tissues from the patients in the discovery cohort using RNA sequencing (Figure 1). Additionally, we examined 1,463 plasma proteins from all study participants (*n* = 330) using the Olink Explore 1536 proximity extension assay.^22^

### Transcriptome signatures differentiate advanced fibrosis

To elucidate the molecular changes associated with advanced hepatic fibrosis, we first analyzed the liver transcriptomics data from patients in the discovery cohort. The samples were grouped based on their histologically assessed fibrosis stages as early stages of fibrosis (stage 0/1/2, referred to as the F0–2 group in subsequent analyses), severe fibrosis (stage 3, F3), and cirrhosis (stage 4, F4) (Figure 2A).

Uniform manifold approximation and projection (UMAP) of the data revealed apparent shifts correlating with the severity of fibrosis (Figure 2B). The samples seemed not segregated according to their gender, lifestyle (e.g., smoking), or comorbidities (e.g., diabetes) (Figures S1A–S1C). When comparing gene expression profiles between advanced (F3 or F4) and early stages (F0–2) of fibrosis (Data S1), gene set enrichment analysis identified significant upregulation of pathways related to ECM remodeling and cell-matrix interaction (e.g., ECM-receptor interaction, focal adhesion, and regulation of actin cytoskeleton) (Figure 2C; Data S2). In line with previous findings demonstrating inflammation-featured fibrosis progression,^5^ our data further emphasized that pathways related to immune response and inflammation (e.g., hematopoietic cell lineage and interleukin [IL]-17, tumor necrosis factor, phosphatidylinositol 3-kinase-Akt, and p53 signaling pathways) were upregulated in advanced fibrosis (Figure 2C). Conversely, metabolic pathways linked to peroxisome, carbon, and energy metabolism were downregulated in cirrhosis (Figure 2C). Some of these pathways showed significant downregulation from F2 onward in patients with MASLD (Figure S2), underlying metabolic dysfunction as a key hallmark of progressive MASLD. Notably, transcription factor enrichment analysis revealed the upregulation of transcriptome targeted by *NF-κB1*, *REL*, *RELA*, *JUN*, *JUND*, *ATF4*, *SMAD3*, *CREB1*, and *PARP1* in advanced fibrosis (Figure 2D). These transcription factors, which are key regulators of inflammation, immune response, and response to cellular stress, have been implicated in the development and progression of fibrosis.^23^^,^^24^^,^^25^^,^^26^^,^^27^

In addition, differential gene expression analysis identified 93 differentially expressed genes (DEGs; adjusted [adj.] *p* < 0.05) in the F3 stage of fibrosis compared to the F0–2 group. Over 97% of these genes also exhibited significance in cirrhotic livers, with a total of 8,328 DEGs identified (Figure 2F; Data S1). This DEG set includes markers of myofibroblasts in the liver,^17^ such as liver-resident activated hepatic stellate cells (e.g., *ACTA2*, *COL1A1*, *SPP1*, and *PDGFRB*), markers associated with epithelial-to-mesenchymal transition (e.g., *KRT19*), activated portal fibroblasts (e.g., *CALCA*, *COL15A1*, *THY1*, and *IL-18R1*), and bone-marrow-derived fibrocytes (e.g., *CXCR4* and *ICAM1*) and mesenchymal stem cells (e.g., *MCAM*) (Figure 2F; Data S2).

To further investigate whether the extensive transcriptional alterations that occur in the cirrhotic liver correlate with specific etiologies, we separately analyzed the cirrhotic samples from patients with ARLD, MASLD, and CVH. The transcriptome profiles did not exhibit clear distinctions among these etiology-based groups in the study (Figure 2G). One gene, *TBC1 domain family member 3*, was significantly overexpressed in the patients with MASLD compared to those with ARLD. A total of 22 and 501 genes showed significant changes in ARLD versus CVH and MASLD versus CVH, respectively, with 17 genes related to viral infection and immune response being shared between the two comparisons (Figure 2H; Data S3). Interestingly, functional analysis of DEGs in MASLD versus CVH highlighted a significant enrichment (adj. *p* < 0.05) in biological processes related to nucleosome assembly, chromatin organization and remodeling, DNA packaging, and the regulation of viral processes (Figure S1D; Data S3). These results may suggest that host-virus interactions are crucial in the pathogenesis of cirrhosis resulting from viral infection, compared to cirrhosis associated with metabolic dysfunction.

### Single-cell deconvolution reveals cell type composition heterogeneity and its association with fibrosis score

**Figure 3:**
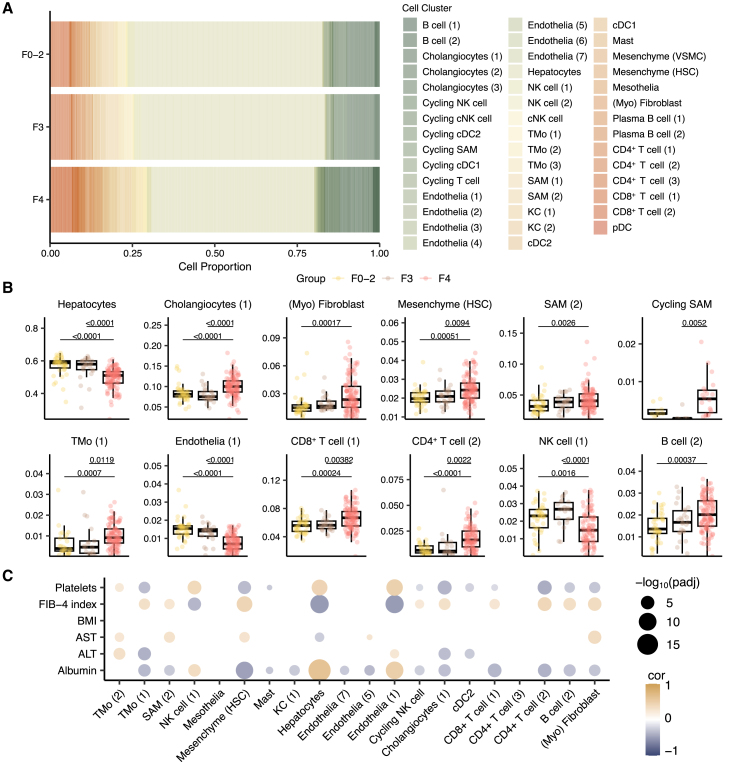
Single-cell deconvolution reveals heterogeneity in cell type composition and its association with fibrosis score (A) Barplot showing the estimated cell population from bulk RNA sequencing data from hepatic tissues. (B) Boxplots showing the significantly differentiated cell populations in groupwise comparisons. Adjusted *p values* were derived from Kruskal-Wallis’s test followed by the Dunnett post hoc pairwise test. The boxplots represent the interquartile range (IQR), with the horizontal line indicating the median. (C) Spearman coefficient correlation between the estimated cell proportion and clinical measurements. The size and color of the dots are proportional to the statistical significance indicated by the negative log_10_ of adjusted *p* values using Benjamini and Hochberg false discovery rate and correlation coefficient, respectively. Adjusted *p* value < 0.05 was considered as statistical significance. Abbreviations: KCs, Kupffer cells; pDCs, plasmacytoid dendritic cells; HSCs, hepatic stellate cells; VSMCs, vascular smooth muscle cells; cDCs; conventional dendritic cells; SAMs, scar-associated macrophages; TMo, tissue monocytes; cNKs, cytotoxic natural killer cells.

A previous study using single-cell RNA sequencing has shed light on the heterogeneity in cell populations within the fibrotic niche in patients with advanced cirrhosis.^11^ To explore the cellular composition changes within the liver microenvironment across different stages of fibrosis, we next performed single-cell deconvolution using a dampened weighted least squares algorithm.^28^ This approach computationally estimates the cell type proportions from bulk gene expression data. We computed and analyzed a total of 44 cell populations annotated by Ramachandran et al.^11^ and Duan et al.^29^ to identify fibrosis-dependent differences. We also assessed the correlation between the abundance of these cell populations and several key clinical variables, including body mass index (BMI) and fibrosis score (fibrosis-4 [FIB-4]). The FIB-4 was calculated based on age and the plasma levels of aspartate aminotransferase (AST), alanine aminotransferase (ALT), and platelet count (STAR METHODS; Figure 3).

We identified 12 cell (sub)populations that exhibited significant abundance change among the fibrosis-dependent groups (adj. *p* < 0.05; Figures 3A and 3B). The pairwise analysis revealed that hepatocytes, CD34^−^CLEC4M^+^ liver sinusoidal endothelial cells, and natural killer cells had significantly lower abundance in cirrhosis (F4 group) compared to early stages of fibrosis (Figures 3A and 3B). Additionally, the scar-associated macrophages (SAMs) differentiated by markers such as *TREM2*, *CD9*, *SPP1*, *TNFSF12*, and *LGALS3* and *PDGFRA*^+^ myofibroblasts had an increased proportion in patients with cirrhosis (Figure 3B). These observations align with the expression patterns of these marker genes in patients with different stages of fibrosis (Figure S3). It has been reported that injury to epithelial cells can promote the release of pro-inflammatory mediators, triggering the activation and differentiation of T cells, including Th1, Th2, and Th17.^5^^,^^30^ In line with this, we observed that two CD4^+^ and CD8^+^ T cell subpopulations differentiated by markers like *SELL*, *CCR7*, and *CD8A* had significant expansion in the cirrhotic livers (Figure 3B). Notably, the abundance of these expanded cell types positively correlated with fibrosis scores across the cohorts (Spearman *r* > 0.25; adj. *p* < 0.05; Figures 3B and 3C). Overall, these results indicate a heterogeneous microenvironment in the liver and offer valuable insights into the alterations in the abundance of various cell types depending on the fibrotic stages and the extent of inflammation across different stages of fibrosis.

### Plasma proteomic changes in patients with liver disease reflect disease severity

**Figure 4:**
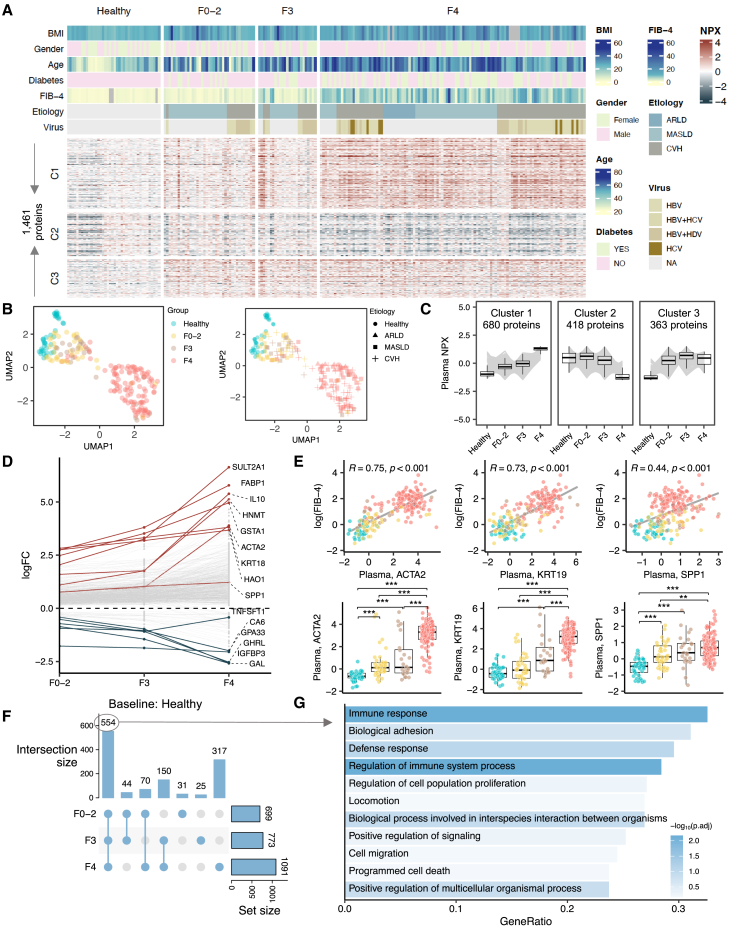
Plasma proteomic changes in patients with liver disease reflect disease severity (A) Plasma proteins profiling of subjects in the discovery cohort. The rows of heatmap were split based on the fuzzy cluster to which a protein belongs to. The columns of heatmap were split based on the group to which a sample belongs to. The column is annotated based on clinical and biochemical parameters of the sample. B) UMAP analyses were performed on the proteome obtained from subjects in the discovery cohort. Each data point represents a sample in the respective colored group. (C) Fuzzy clustering identified three protein clusters with distinct abundance patterns corresponding to disease severity. The individual gray line represents the median abundance of individual proteins in the cluster, and the boxplot represents the median, quartile values for all proteins in the cluster across different groups. (D) Log transformation of fold change (logFC) of top 5 proteins in each groupwise comparison. (E) Spearman correlation between fibrosis markers (*ACTA2*, *KRT19*, and *SPP1*) and fibrosis score (fibrosis-4, FIB-4) (upper) and their plasma levels across groups (lower). ∗adjusted *p* value < 0.05, ∗∗adjusted *p* value < 0.01, ∗∗∗adjusted *p* value < 0.001 derived from DESeq2. The boxplots represent the interquartile range (IQR), with the horizontal line indicating the median. (F) Upset plot summarizing the numbers of proteins with levels significantly different in patients compared to healthy subjects. (G) The biological processes enriched in the common set of differentially expressed proteins across comparisons.

To investigate the impact of liver pathology on plasma proteome and its potential to reflect liver fibrosis, we further characterized the plasma proteome profiles from both healthy individuals and patients with different stages of hepatic fibrosis. UMAP analysis revealed a clear separation between the proteome profiles of healthy individuals and those of the patients. Within the patient groups, we observed gradual shifts in proteome profiles correlated with advancing stages of fibrosis in the liver (Figures 4A–4C, S4A, and S4B). To illustrate whether the proteome changes indicate biological processes being altered as the disease progresses, we used Mfuzz clustering^31^ to group proteins with similar abundance changes and identified three clusters of protein trajectories across fibrosis-dependent groups (Figures 4A, 4C, and S4C; Data S4). Specifically, 680 proteins in cluster 1, including pro- and anti-inflammatory cytokines (e.g., *IL*-*6* and *IL*-*10*) and liver fibrosis markers (e.g., *ACTA2*, *KRT19*, and *SPP1*), increased progressively with fibrosis stages (Figures 4D and 4E). 418 proteins within cluster 2, associated with wound healing and vesicle-mediated transport, exhibited a slight increase in median levels in the early stages of fibrosis, followed by an acute decrease in cirrhosis. Additional 363 proteins in cluster 3 that were largely immune response and signaling regulation related were consistently elevated in patients with liver fibrosis (Figure S3C).

In parallel, we analyzed the levels of proteins at different stages of liver fibrosis compared to healthy controls. Our findings showed significant (adj. *p* < 0.05) changes of 699, 772, and 1,091 proteins in the F0–2, F3, and F4 groups, respectively (Figures 4F, S5, and S6; Data S5). Among these, 554 proteins were differentiated in common across all diseased groups. Approximately 33% of these proteins were significantly enriched in immune response (adj. *p* < 0.05; Figure 4G), including elevated levels of chemokines (e.g., *CCL3*, *CCL4*, *CCL11*, *CXCL6*, *CXCL13*, and *CCL24*), cytokines (e.g., *OSM*, *IL*-*6*, *IL*-*10*), growth factors (e.g., *TGFB1* and *MDK*), and tumor necrosis factor receptors (e.g., *FAS*, *TNFRSF1B*, *TNFRSF4*, *TNFRSF11A*, and *TNFRSF14*) (Data S5). For instance, *IL*-*10*, a key anti-inflammatory cytokine, was found to be one of the most significantly elevated proteins in patients (Figure 4D). The elevated *IL*-*10* has been previously implicated in the pathophysiological process of immune cell paralysis that characterizes the dysfunctional immune response in acute-on-chronic liver failure.^32^ Additionally, biological adhesion was identified as the second most overrepresented category (Figure 4F). Specifically, we observed an upregulation in proteins associated with adhesion junctions, including E-cadherin (*CDH1*), N-cadherin (*CDH2*), and protocadherin 1. Proteins involved in focal adhesions, such as paxillin and integrin alpha 5, were also found to be upregulated in the plasma samples of patients (Data S5).

We also investigated proteome alterations associated with different etiologies in patients with cirrhosis. In comparing the protein profiles of patients with ARLD and MASLD, we found no proteins exhibiting significant differences in their plasma levels. Between the patients with ARLD and CVH, we identified 24 proteins (e.g., *GSTA3*, *DCXR*, *FBP1*, and *ALPP*) that exhibited significant differences in plasma levels (Figure S4D), while their hepatic mRNA expression levels were not differentially expressed between the groups (Figure S4E). Similarly, among the 501 DEGs identified between the livers of patients with MASLD and CVH, in the liver, with 49 of their corresponding proteins being measured in plasma, none had significance in plasma levels between the patients with MASLD and those with viral hepatitis.

### Proteo-transcriptomic signatures associated with advanced fibrosis

**Figure 5:**
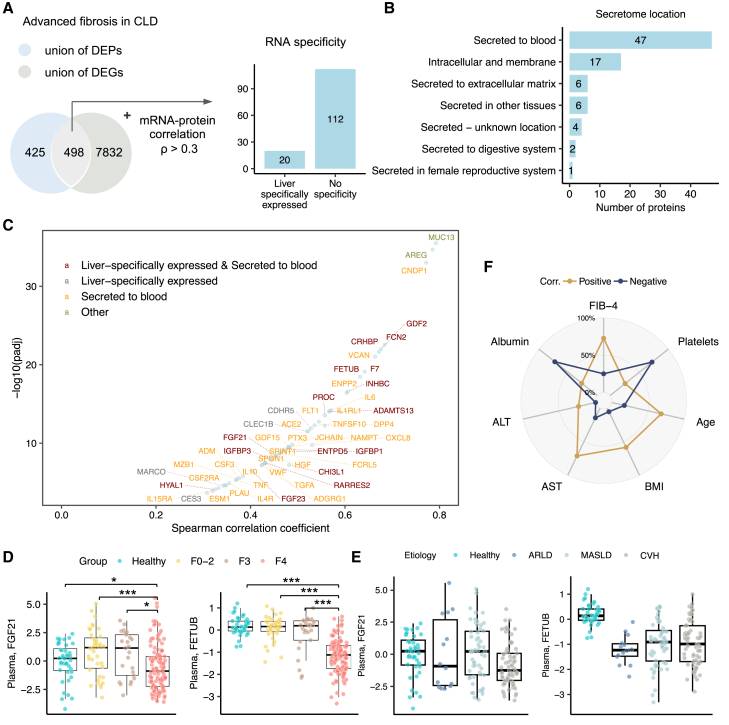
Integrative analysis of liver and plasma omics profiles (A) Venn diagram showing the intersection between the union of DEPs and DEGs associated with advanced fibrosis. (B) The secretome location is predicted for filtered proteins according to the annotation in Human Protein Atlas.^35^. (C) Spearman correlation between the levels of mRNA-protein for filtered proteins. (D and E) (D) Boxplot showing the plasma levels of FGF21 and FETUB in fibrosis-based groups and (E) in etiology-based groups. ∗adjusted *p* value < 0.05, ∗∗adjusted *p* value < 0.01, ∗∗∗adjusted *p* value < 0.001 derived from DESeq2. This boxplots represent the interquartile range (IQR), with the horizontal line indicating the median. (F) Radar plot showing the proportion of proteo-transcriptome signature proteins that are positively or negatively correlated with clinical parameters, including FIB-4, albumin, platelets, AST, ALT, BMI, and age. Abbreviations: DEPs, differentially expressed proteins; DEGs, differentially expressed genes.

We next sought to integrate the proteome and transcriptome signatures associated with advanced fibrosis by performing a pairwise correlation analysis between significantly altered genes and their corresponding protein levels, following approaches recently applied in proteo-transcriptomics studies.^33^^,^^34^ We found that 498 proteins demonstrated significant differences in both hepatic gene expression levels and in their abundance in the plasma in patients with advanced fibrosis compared to those in the early stages (Figure 5A). Notably, the abundance of 132 of these proteins correlated strongly (STAR METHODS; Spearman *r* > 0.3; adj. *p* < 0.05) with their hepatic mean mRNA expression across the patients’ cohort (defined as the proteo-transcriptomic signatures for downstream analyses), including 20 proteins that are of hepatic origin according to the annotation of Human Protein Atlas^35^ (Figures 5A and 5B; Data S6). Moreover, mapping the proteo-transcriptomic signatures onto a subcellular map of human proteins^36^ revealed that 47 of them are secreted into the bloodstream (Figures 5B and 5C). For instance, hepatokine *FGF21*, a stress-inducible hormone mainly expressed and secreted by the liver to the bloodstream to act in an endocrine manner,^37^ demonstrated a positive correlation (*r* = 0.45) between mRNA and protein levels across the patients’ cohort (Data S6). The mRNA-protein level of fetuin B (*FETUB*), a cystatin superfamily protein secreted by the liver, exhibited a strong correlation (*r* = 0.63) in the patient cohort (Figure 5D). A previous study by Meex et al.^38^ demonstrated that the plasma level of *FETUB* increased in obese participants with simple steatosis and was associated with insulin resistance.^38^ Interestingly, we found that the levels of *FETUB* increase in patients with early stages of fibrosis (F0–2) and F3, while significantly decreasing in patients with cirrhosis compared to healthy controls (Figure 5F). This observation aligns with the decreased expression of *FETUB* in hepatocytes of cirrhotic livers at the single-cell resolution (Figure S3B), suggesting a potential role for these hepatokines in reflecting the progression and severity of liver damage. We also explored the correlation between the plasma abundance of the 132 proteins and clinical parameters. As shown in Figure 5F, most of the proteins showed a positive correlation with FIB-4 but a negative correlation with plasma albumin level and platelet counts.

### Biomarker panels for advanced fibrosis and cirrhosis

To further pinpoint proteins capable of stratifying patients with advanced fibrosis based on the proteo-transcriptomic signatures, we developed two binary classifiers aimed at distinguishing (1) patients with advanced fibrosis (stage 3 or higher, referred to as the ≥F3 model) and (2) patients with cirrhosis (the cirrhosis model). For feature selection, we employed a random forest (RF) algorithm, training the models on 70% of the samples using stratified 5-fold cross-validation and built-in parameter tuning in the discovery cohort. The models were constructed to differentiate the case group from a control group, which comprised all other non-case samples in each specific model. The performance of these models was then tested on the remaining 30% of the samples in the same cohort and on an additional validation cohort of 112 patients at different stages of liver disease (Table 1).

**Figure 6:**
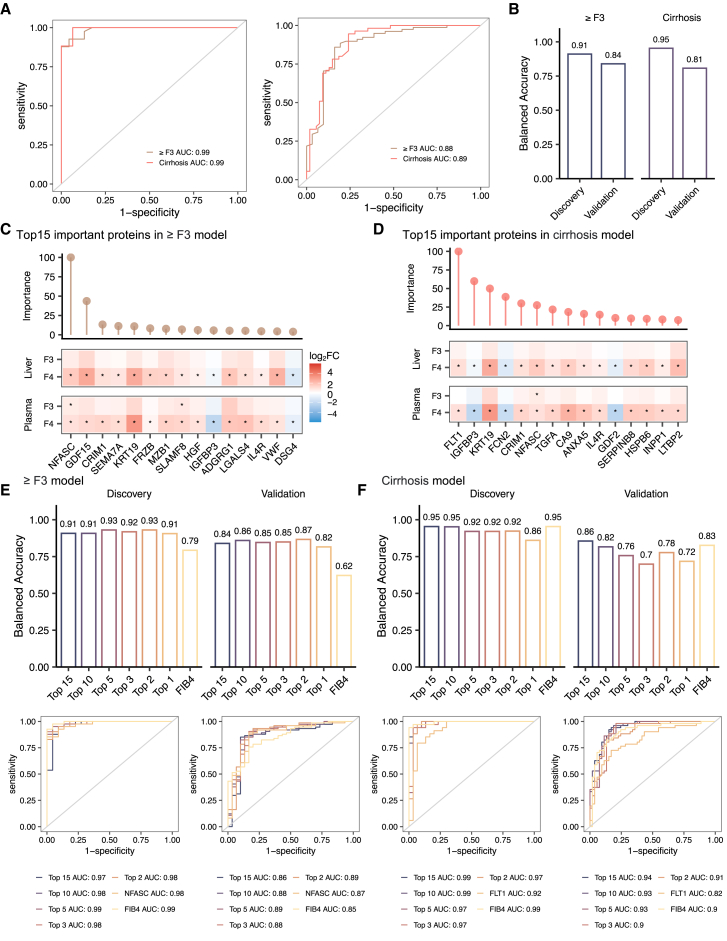
Biomarker panels for advanced fibrosis and cirrhosis (A and B) The AUC-ROC curves (A) and balanced accuracy (B) for prediction of advanced fibrosis (≥F3 model) and cirrhosis (≥F4 model) in discovery and validation cohorts using random forest algorithms based on plasma proteins. (C and D) Top 15 important proteins identified by random forest algorithm from the ≥F3 (C) and cirrhosis (D) models, respectively. The bar on the top of each plot shows the importance of the protein in the respective model. The heatmap on the bottom shows the relative changes (log2 fold change) of hepatic mRNA and plasma protein in advanced fibrosis as compared to those in the F0/1/2 stages. ∗adjusted *p* value < 0.05. (E and F) The AUC-ROC curves and balanced accuracy for the prediction of advanced fibrosis (E) and cirrhosis (F) based on the top 15, top 10, top 5, top 3, and top 2, the first important proteins in both discovery and validation cohorts using logistic regression algorithm.

The final trained RF models had area under the receiver operating characteristic curve (AUROC) scores of 0.98 (95% confidence interval [CI] 0.97–1) and 0.99 (95% CI 0.98–1) for the prediction of ≥ F3 and cirrhosis, respectively, in the discovery cohort (Figure 6A). In the validation cohort, the models achieved AUROC scores of 0.88 (0.80–0.96) and 0.89 (95% CI 0.83–0.96) for predicting ≥ F3 and cirrhosis, respectively (Figure 6A). Both models achieved balanced accuracies up to 91% and 81% in the discovery and validation cohort, respectively (Figure 6B). The importance scores of each protein estimated during model training phase are provided in Data S7, indicating the extent to which a protein is relevant to the classification task. Additional model performance scores are available in Data S7 for each model. In Figures 6C and 6D, we present the top 15 proteins with the highest importance score in each model and their relative changes in the liver and plasma in patients with advanced fibrosis, as compared to those in the early stages. Among the 25 unique proteins in these two models, 3 were “liver specific” and secreted into the bloodstream, including *FCN2*, *IGFBP3*, and *GDF2* (Figures 5C, 6C, and 6D). *NFASC* and growth differentiation factor 15 (*GDF15*) exhibited the highest importance in predicting advanced fibrosis (Figure 6C).

Further analysis using logistic regression yielded comparable predictive power of the top 15 features (proteins) in predicting ≥ F3 and cirrhosis, with AUROC scores of 0.97 (95% CI 0.92–1) and 0.99 (95% CI 0.98–1) in the discovery cohort and 0.85 (95% CI 0.76–0.95) and 0.94 (95% CI 0.89–0.98) in the validation cohort (Figure 6E; Data S7). In addition, we investigated the predictive power of various subsets of these top proteins (e.g., top 1, 2, 3, 5, 10, and 15) and compared them to models trained using FIB-4 (Figure 6E). Our analysis showed that the model trained with *NFASC* and *GDF15* had the superior performance in the prediction of advanced fibrosis, with about 93% and 87% balanced accuracies in the discovery and validation cohorts, respectively, compared to that trained by FIB-4 (79% and 62% balanced accuracies, respectively) (Figure 6E). For the cirrhosis model, the top 10 proteins (*FLT1*, *IGFBP3*, *KRT19*, *FCN2*, *CRIM1*, *NFASC*, *TGFA*, *CA9*, *ANXA5*, and *IL*-*4R*) resulted in balanced accuracies of approximately 95% and 83% in the discovery and validation cohorts, respectively (Figure 6F).

## Discussion

This study combined hepatic transcriptomics, systems-level proteomics, integrated deconvolution analysis, and machine learning models to provide a comprehensive characterization of liver fibrosis in patients with CLD, encompassing CVH, ARLD, and MASLD. Our transcriptomics analysis across fibrosis stages revealed the upregulation of pathways related to ECM remodeling, inflammation, and immune response in advanced fibrosis. This supports previous findings that underscore the role of these pathways in fibrogenesis.^5^^,^^39^

Previously, Govaere et al.^40^ identified 25-gene signatures differentially expressed in the liver during progressive MASLD. Consistent with these findings, our study observed that 24 of these genes showed increased expression in advanced fibrosis within our MASLD subgroups (Figure S2D), including five genes (*STMN2*, *GDF15*, *LTBP2*, *ITGBL1*, and *THY1*) that were differentially expressed in both F3 and F4 stages. Notably, this MASLD-related signature also exhibited similar expression patterns in our whole cohort analysis, suggesting that these genes may also play significant roles in fibrosis progression across different CLDs, including ARLD and viral hepatitis. For instance, Luan, Harding H. et al. demonstrated that *GDF15*, part of the transforming growth factor β superfamily, is induced during bacterial and viral inflammation and promotes metabolic adaptations to systemic inflammation.^41^ In our study, we identified 132 proteo-transcriptomic signatures associated with advanced fibrosis. Alongside this set, our results revealed transcriptomic differences driven by specific underlying causes, including significant changes in genes involved in host-virus interactions in patients with viral hepatitis.

Ramachandran et al.^11^ provided insights into the cellular and molecular basis of human liver cirrhosis (F4) at a single-cell level. A key strength of our deconvolution analysis is that it elucidates the dynamic changes in cell populations—either expanding or declining—that have altered in cirrhotic livers, starting from early fibrosis stages, thereby enhancing our understanding of the pathophysiological processes driving fibrosis progression.^42^ Hepatocellular injury during fibrosis is well documented. Additionally, Ramachandran et al.^11^ observed that SAMs accumulate in the scar tissue of cirrhotic livers and stimulate the production of fibrillar collagen, further contributing to fibrosis development. Our study’s predicted cell type proportions are consistent with the presence of SAMs in cirrhosis. Importantly, our data indicate that SAM expansion is associated with the severity of fibrosis caused by different etiology. Moreover, we noted immune cell expansions as fibrosis progressed, suggesting a potential role for the immune system in driving or responding to fibrotic changes. Collectively, our analysis highlighted significant alterations in cellular composition, transitioning from early fibrosis to advanced fibrosis and cirrhosis.

A promising precision medicine approach to complex disease diagnosis and management involves the use of protein profiling to capture the disease processes occurring in tissues more effectively.^43^ When combined with histological data and liver transcriptomics from well-characterized cohorts, our results show extensive remodeling of the plasma proteome correlated with progressive fibrosis. For instance, we observed significant dysregulation of proteins involved in inflammation and immune responses across different stages of liver fibrosis in patients with CLD. This supports the hypothesis that systemic inflammation plays a critical role in the development of liver fibrosis.^5^ Additionally, our findings enhance our understanding of the molecular pathophysiology underlying liver fibrosis and demonstrate the feasibility of using the plasma proteome for more accurate prediction of liver diseases. The combined machine learning models achieved higher balanced accuracy with the proposed biomarker panels for predicting advanced fibrosis and cirrhosis compared to established clinical markers. This underscores the potential for improving the early diagnosis of advanced fibrosis in patients with CLD through plasma protein profiling.

In summary, through multi-omics approaches, we have illustrated transcriptional dysfunction and cellular composition dynamics depending on fibrosis stages in patients with CLD from various causes. The identification of circulating protein signatures further contributes to our understanding of the pathogenesis and potential early diagnosis of liver fibrosis.

### Limitations of the study

While our study has provided detailed molecular insights into advanced fibrosis in CLD, selected limitations are highlighted here. First, the cohort composition primarily includes patients with advanced fibrosis and cirrhosis, limiting the applicability of the findings to earlier stages of liver disease such as significant fibrosis (fibrosis stage ≥2). Future studies should include a broader range of fibrosis stages and larger cohorts stratified by fibrosis etiology to determine the molecular differences associated with fibrosis severity and etiology in patients with CLD. Second, given the complexity and breadth of the human plasma proteome, the proteomics assay used in the study may not capture all relevant proteins involved in liver fibrosis and its progression. Using additional or alternative omics technologies, such as mass spectrometry, could provide a more comprehensive understanding of diseases in future studies. Third, while our findings suggest potential protein biomarkers for the prediction of advanced fibrosis in CLD, the extent to which they apply in the general population remains to be fully elucidated in future large cohort studies, especially in less represented etiology subgroups. Lastly, the cohorts used in the study are from specific geographic regions and ethnic backgrounds; further studies with a wide age range are warranted to validate and extend the findings across different populations.

## Resource availability

### Lead contact

Further information and requests for resources and reagents should be directed to and will be fulfilled by the lead contact, Dr. Mujdat Zeybel (mzeybel@ku.edu.tr).

### Materials availability

This study did not generate new unique reagents.

### Data and code availability

• All the data generated or analyzed during this study are included in this published article and/or the supplemental information. The raw transcriptomics data have been deposited at GEO (accession number GEO: GSE276114) and are publicly available as of the date of publication. The plasma proteomics data are available from Mendeley Data at https://doi.org/10.17632/6brkvh3f97.1.
• This paper does not report original code. We used publicly available software/R packages in all the analyses. These are listed with appropriate citations in the methods.
• Any additional information required to reanalyze the data reported in this is available from the lead contact upon request.

## Acknowledgments

The authors would like to acknowledge financial support from ScandiEdge and ScandiBio Therapeutics and the 10.13039/501100004063Knut and Alice Wallenberg Foundation (no. 72110). A.M. and H.Y. acknowledge support from the PoLiMeR Innovative Training Network (Marie Skłodowska-Curie grant agreement no. 812616), which has received funding from the European Union’s Horizon 2020 research and innovation program. The authors gratefully acknowledge the use of the services and facilities of the Koç University Research Center for Translational Medicine (KUTTAM), equally funded by the Republic of Turkey Ministry of Development Research Infrastructure Support Program. Findings, opinions, or points of view expressed in this article do not necessarily represent the official position or policies of the Ministry of Development. This project was supported by the Horizon 2020 Marie Curie Sklodowska Individual Fellowship (to M.Z.) and 10.13039/501100004410Scientific and Technological Research Council of Turkey (TUBITAK) 1001 grant with a project number of 117S440 (to M.Z.). The computations were performed on resources provided by SNIC through the 10.13039/501100015701Uppsala Multidisciplinary Center for Advanced Computational Science (UPPMAX) under project sllstore2017024, sctatlas, and naiss2023-6-92. The graphical abstract was created with BioRender.com.

## Author contributions

A.M. and M.Z. conceived and designed the study; H.Y., M.Y., M.L., M.B.A., L.M., and C.Z. performed data analysis and interpretation; D.A. and E.D. collected clinical data; D.A., O.A., I.B.P., B.U., B. Yigit, T.S., and B. Yüksel collected biological samples; C.Y., M.A., and M.D. recruited studying cohorts; H.K. and B.S. performed histopathological analysis; and H.Y. drafted the manuscript. All authors read and approved the final manuscript.

## Declaration of interests

A.M., J.B., and M.U. are founders and shareholders of ScandiEdge and ScandiBio Therapeutics.

## STAR★Methods

### Key resources table

**Table:** 

REAGENT or RESOURCE	SOURCE	IDENTIFIER
**Biological samples**		

Human blood from healthy adults and patients	This paper	N/A
Human liver samples	This paper and Ramachandran et al.^11^	N/A

**Critical commercial assays**		

Olink Explore 384 Cardiometabolic Reagent Kit	Olink	Panel lot number: B04413, Product number: 97700/97300
Olink Explore 384 Inflammation Reagent Kit	Olink	Panel lot number: B04411, Product number: 97500/97100
Olink Explore 384 Oncology Reagent Kit	Olink	Panel lot number: B04412, Product number: 97600/97200
Olink Explore 384 Neurology Reagent Kit	Olink	Panel lot number: B04414, Product number: 97800/97400
Qiagen RNeasy mini kit	QIAGEN, US	Catalog No.74104
RNeasy micro kit	QIAGEN, US	Catalog No.74004

**Deposited data**		

Raw and processed bulk RNA-sequencing data	This paper	GEO: GSE276114
Proximal extension assay proteomic dataset	This paper	Mendeley Data: https://data.mendeley.com/datasets/6brkvh3f97/1
Single cell RNA-sequencing data	Ramachandran et al.^11^ and Duan et al.^29^	Original data: GSE136103; Processed/annotated data available from https://datashare.ed.ac.uk/handle/10283/3433^2^

**Software and algorithms**		

Rstudio v2023.09.0 + 463	Rstudio Team	http://www.rstudio.com/
R software v4.3.3	R CRAN	https://www.r-project.org/
ComplexHeatmap R package v2.18.0	Gu et al.^44^	https://bioconductor.org/
ggthemes R package v5.1.0	Arnold et al.^45^	https://github.com/jrnold/ggthemes
limma R package v3.58.1	Ritchie et al.^46^	https://bioconductor.org/
clusterProfiler R package v4.11.0	Yu et al.^47^	https://bioconductor.org/
msigdbr R package v7.5.1	Bhuva D et al.^48^	https://bioconductor.org/
Mfuzz R package v2.58.0	Kumar and M^31^	https://bioconductor.org/
NbClust R package v3.0.1	Charrad et al.^49^	https://cran.r-project.org/web/packages/NbClust/index.html
caret R package v6.0.94	Kuhn et al.^50^	https://topepo.github.io/caret/
pROC R package v1.18.5	Robin et al.^51^	https://xrobin.github.io/pROC/
DESeq2 R package v1.34.0	Love et al.^52^	https://bioconductor.org/
Kallisto R package v0.46.2	Bray et al.^53^	https://pachterlab.github.io/kallisto/
tximport R package v1.22.0	Soneson et al.^54^	https://bioconductor.org/
ggplot2 R package v3.5.0	Wickham et al.^55^	https://ggplot2.tidyverse.org/
DWLS R package	Tsoucas et al.^28^	https://github.com/dtsoucas/DWLS
Seurat R package v4.3.0	Satija et al.^56^	https://satijalab.org/seurat/

### Experimental model and study participant details

#### Participant information: Ethical approval and patient consent

This study received approval from the Ethics Committees of Koç University in Istanbul. Before clinical sample and data collection, patients were informed, and their written consent was taken, ensuring that ethical guidelines were followed across all participating centers (2015.053.IRB1.014, 2016.024.IRB2.005, 2017.139.IRB2.048, 2022.246.IRB2.040). As this work does not involve a clinical trial, no clinical registry numbers or associated links are applicable.

#### Participant information: Inclusion and exclusion criteria

The study included adults aged 18–80 years who can provide informed consent and have a confirmed diagnosis of chronic liver disease through clinical evaluation, imaging, and histopathological findings. General exclusion criteria encompassed the presence of acute liver disease and other chronic liver diseases, such as Budd-Chiari syndrome, Wilson’s disease, autoimmune hepatitis, and drug-induced liver disease. Additional exclusion criteria include concurrent severe systemic illnesses, such as advanced cardiovascular disease, sepsis, or any malignancy other than HCC. Participants who were pregnant or lactating and had a history of organ transplantation were also excluded. For patients with MASLD and ARLD, diagnosis-specific inclusion and exclusion criteria are determined following guidelines outlined by Rinella et al., 2023 2. For those with CVH, inclusion criteria involve a diagnosis of chronic viral hepatitis confirmed by serological tests. Patients diagnosed with hepatocellular carcinoma (HCC) met the general background etiology criteria of MASLD, ARLD, or CVH and had histologically or radiologically confirmed HCC.

#### Participant information: Sample size estimation

*A priori* power analysis was conducted to determine the minimum sample size required to detect statistically significant proteomic and transcriptomic changes associated with advanced fibrosis. The analysis used a t-test for the difference between two independent means (two groups). The test was set up with two tails, assuming an effect size (Cohen’s d) of 0.5, a significance level (α) of 0.05, and a desired power (1-β) of 0.80. An allocation ratio of 7:1 (N2/N1) was used because of the differences in group sizes. Based on these parameters, the calculated total sample size was 290 participants, with 36 participants required in group 1 (healthy patients) and 254 in group 2 (CLD patients). This calculation produced a non-centrality parameter (δ) of 2.8076, a critical t-value of 1.9682, and an actual power of 0.7991. This analysis provided us with the sample size that was adequately powered to detect differences in molecular profiles across fibrosis stages.

#### Participant information: Experimental group allocations

Subjects and samples were allocated to experimental groups based on clinical diagnoses and fibrosis stages. The study was divided into a discovery cohort (*n* = 218) and a validation cohort (*n* = 112). The discovery cohort included 40 healthy controls and 178 chronic liver disease (CLD) patients, while the validation cohort comprised 112 CLD patients to validate the differential proteomic profile of advanced fibrosis in the discovery group. CLD patients were stratified into fibrosis stages based on histological evaluation as early fibrosis (F0-2), severe fibrosis (F3), and cirrhosis (F4). Fibrosis staging was determined using the METAVIR scoring system for CVH and the Kleiner system for MASLD. Patients were further categorized by their chronic liver diagnosis by clinical experts of hepatology. Healthy controls were matched to CLD patients and derivation cohort patients were matched to the validation cohort patients for demographic factors.

### Method details

#### Clinical data collection

Comprehensive clinical data were gathered from the clinical portals of Koç University Hospital and Medipol University Hospital. The fibrosis stage in the peritumoral liver tissue of HCC patients and liver tissue of CLD patients collected at fasting state were histologically evaluated by expert liver pathologists based on Kleiner et al.^21^ system for MASLD and the METAVIR (meta-analysis of histological data in viral hepatitis) scoring systems^57^ for CVH.

#### Plasma sample collection

Blood samples were collected into EDTA blood collection tubes from healthy adults and patients at Medipol University Hospital and Koç University Hospital for proteomics analysis. To isolate cell-free plasma, the blood samples were centrifuged at 3000 rpm for 10 min at 4°C to separate plasma from the cellular components. The supernatant was then carefully transferred to a new falcon tube without disturbing the buffy coat formed between the pellet and the plasma. For further purification and removal of any remaining cellular components, a second centrifugation step was performed under the same settings as the first centrifugation. The isolated cell-free plasma was then aliquoted and stored at −80°C until proteomics analysis.

#### Liver tissue procurement

For transcriptomics analysis, RNAs were extracted from the explant liver, and biopsy specimens were procured from the patients with MASLD or HCC diagnosis. Liver samples were obtained from patients who had liver transplantation, liver resection surgery, or diagnostic percutaneous core biopsy procedure at Koç University Hospital or Medipol University Hospital. For peritumor HCC samples, the samples were collected at least 1 cm distant from the tumor. The samples were immediately snap-frozen in Qiagen RNAprotect Tissue Reagent (Catalog No. 76106) to ensure RNA integrity. Tissues were stored at −80°C until RNA isolation.

#### RNA extraction and library preparation for transcriptome sequencing

Samples were homogenized using Stainless Steel Beads (Catalog No. 69989) and a Qiagen TissueLyser II system, operating at a frequency of 20 Hz for 4 min. To ensure thorough homogenization, the samples were further processed using Qiagen QIAShredder (Catalog No. 79656). RNA of the tissue homogenates was extracted by Trizol/Chloroform phase separation technique, then RNA purification was carried out with Qiagen RNeasy mini kit for explant liver samples (Catalog No.74104) and RNeasy micro kit (Catalog No.74004) for biopsy samples. After the evaluation of the sample A260, A260/A280, and A260/A230 values of the samples, the RNA integrity number (RIN) value of total RNA samples was measured with TapeStation (Agilent Tech, USA). The amount of total RNA was measured more precisely fluorometrically with the RNA Broad Range kit (Thermofisher, USA) by using Qubit (Invitrogen, USA). The RNA sequencing library was prepared with Illumina Stranded Total RNA Prep and Ligation with Ribo-Zero Plus kit was used following the standard protocol provided by the manufacturer. The libraries were then pair-end (2′100bp) sequenced on the NovaSeq6000 system yielding, on average, 25 million fragment reads per sample. Raw sequencing data (.bcl) was converted to FASTQ with the Dragen Bio-IT platform (v3.9.5). The quality of RNA-seq data was assessed by FastQC (v0.11.9).

#### RNA-seq data processing, differential expression, and functional enrichment analysis

Tissue bulk RNA-seq data were aligned and quantified using a standard protocol of Kallisto (v0.46.2)^53^ against the Human genome GRCh38 (ensembl release 102) downloaded from Ensembl official website (https://www.ensembl.org/index.html). The output of Kallisto, both estimated counts and TPM (transcript per kilobase million)-based transcript-level expressions were then transformed into gene-level expressions using the Bioconductor package tximport (v1.22.0)^54^ with the tx2gene option set to connect transcripts to genes. Protein-coding genes were considered for the above step and downstream analyses. Differential analysis was performed using the DESeq2 R package (v1.34.0),^52^ following a standard protocol for all the pairwise comparisons. Significantly expressed genes (DEGs) were identified with a significance threshold of an adjusted *p*-value <0.05. To examine differences in global gene expression, we conducted principal component analysis (PCA) based on TPM and/or variance-stabilizing transformation (VST)-normalized count data in DESeq2. Uniform manifold approximation and projection (UMAP) dimensionality reduction was used for the visualization of 80% variance calculated by PCA analysis.

*Gene Set Functional Enrichment Analysis*. We used the clusterProfiler R package (v4.11.0)^47^ to perform gene set enrichment analysis against the curated gene sets obtained from the KEGG pathway database or TRRUST.^58^ Benjamini-Hochberg (BH)–adjusted *p*-value <0.05 are considered statistically significant and are provided in the relevant figures/datasets.

#### Cell-type deconvolution based on DWLS

The deconvolution algorithm dampened weighted least squares (DWLS)^28^ was employed in computing the cell proportion from bulk tissue RNA-seq data. The DWLS uses a reference cell type-specific gene expression profile and a list of signature genes specific for the cell types to calculate the cell type proportion. The processed/annotated single-cell data that was originally published by Ramachandran et al.^11^ (available on the online database under accession number GSE136103), were downloaded from the website provided by Duan et al.^29^ Signature genes for each cell type were analyzed using the *FindAllMarkers* function from the Seurat R package (v4.3.0).^56^ The transcript per million (TPM) of marker genes from the liver samples was inputted to DWLS along with signature reference to estimate the cell proportion. The results were analyzed using Kruskal-Wallis analysis of variance followed by Dunnet’s post hoc test.

#### Plasma proteomic profiling and data pre-processing

The levels of 1463 proteins were measured using the Olink Explore 1536 Proximity Extension Assay.^22^ The proteins were presented from four Olink panels: the Olink Explore 384 Cardiometabolic Reagent Kit (Panel lot number: B04413, Product number: 97700/97300), the Olink Explore 384 Inflammation Reagent Kit (Panel lot number: B04411, Product number: 97500/97100), the Olink Explore 384 Oncology Reagent Kit (Panel lot number: B04412, Product number: 97600/97200), and the Olink Explore 384 Neurology Reagent Kit (Panel lot number: B04414, Product number: 97800/97400). In the Olink Explore platform, massively parallel sequencing is used as a readout.

The Olink workflow is detailed in Álvez et al.^59^ In short, antibodies targeting 1463 proteins are first conjugated with two complementary probes and distributed in 384-plex panels. The samples are incubated with the antibodies overnight to allow binding to the corresponding targets and hybridization of the oligos, and are further extended, pre-amplified, amplified and indexed by PCR, and sequenced using Illumina’s NovaSeq platform. Olink’s quality control and normalization pipeline is applied to the resulting counts, which are later transformed into a relative log2 scale unit, the Normalized Protein eXpression (NPX). All protein measurements reported to fail quality control are excluded from the dataset.

#### Differential expression and functional analyses of plasma proteome

The differential protein expression was performed using the limma R package (v3.58.1)^46^ after excluding proteins with missing values in >50% of samples. Proteins with false discovery rate (FDR)-adjusted *p*-values <0.05 are considered statistically significant. The adjusted *p*-values and differences in average expression per group were summarized in volcano plots for each of the pairwise comparisons. Enrichment analysis of protein sets was performed using the clusterProfiler R package (v4.11.0). The *enricher* function in the clusterProfiler package was used to perform overrepresentation analysis against biological process gene sets from MSigDB retrieved using R package msigdbr (v7.5.1),^48^ with subsequent *p*-value adjustment using the Benjamini-Hochberg method. The 1463 proteins analyzed in the study were used as a background list for enrichment/overrepresentation analysis.

#### Fuzzy c-mean clustering of plasma proteome

To reveal protein abundance patterns as the disease advanced, we used a fuzzy c-means clustering approach to perform pattern recognition using the ‘Mfuzz’ R package (v2.58.0)^31^ after normalization of the data. We first used the NbClust method, available in the NbClust R package (v3.0.1),^49^ to determine the optimal number of clusters of proteins with similar patterns. In this study, the number of clusters was chosen based on the support from the most NbClust testing methods (Euclidean distance, Ward method clustering, searching from 2 to 10 clusters).

#### Pairwise liver-plasma correlation analysis

Pairwise correlation was performed to assess the relationship between mean gene expression in the liver and its end-product protein levels in the paired plasma across the patient cohort. The Spearman coefficient of the gene-protein pair was assessed using the *cor.test* function in the R stats package (v4.3.3). The significance level was controlled at an FDR-adjusted *p*-value (adj.p) by Benjamini–Hochberg of <0.05 and a value of correlation coefficient r > 0.3. Significant proteins and corresponding *p*-values, Spearman correlation coefficients and annotations from the Human Protein Atlas are provided in Data S6.

#### Machine learning models for advanced fibrosis and cirrhosis

Two classification models were constructed to 1) differentiate patients with advanced fibrosis (≥F3), and 2) patients with cirrhosis (F4), respectively, using the caret R package (v6.0.94).^50^ The data in the discovery cohort were split into 70% as a training set and 30% for a testing set using the *createDataPartition* function in caret, generating a training and testing pool of samples. Before the model training, the data with missing values due to failed quality control were imputed using the *preProcess* function in caret with the “*knnImpute*” method. Highly correlated proteins were removed using the *findCorrelation* function (Pearson’s correlation *r*^*2*^ > 0.9). The models first were built on the training sets using the *train* function in caret, and a random forest algorithm was used as the classification algorithm for feature selection. We fine-tuned the hyperparameters of each model using a 5-fold cross-validation strategy on the training set and evaluated the best models on the test sets, including the remaining 30% of the samples in the same cohort and an additional cohort. The *predict* function in the caret package was used to evaluate model performance. The area under the receiver operating characteristic curve (AUROC) scores and balanced accuracy were used as performance metrics. The overall importance of each protein, with values ranging between 0 and 100%, was estimated during the model training phase and extracted using the *varImp* function in the caret package, indicating the extent to which a protein is relevant to the classification task. The 95% confidence intervals of AUC were calculated by the DeLong test^60^ implementation in the pROC R package (v1.18.5).^51^ For further evaluation of the predictive power of selected proteins in each model, we used the logistic regression algorithms in the *train* function in the caret package.

### Quantification and statistical analysis

To summarize the patient characteristics, descriptive statistics were employed. Continuous variables were evaluated for normality by using the Shapiro-Wilk test. Normally distributed variables were summarized with mean ± standard deviation (SD) while non-normally distributed variables were described using medians and interquartile ranges. Categorical variables were represented through frequencies and percentages. The formula for calculation of FIB-4 index: FIB-4 = Age(years)×AST (U/L)/[PLT(10ˆ9/L)×ALTˆ(½) (U/L)]. Differences between multiple groups were tested by ANOVA or a non-parametric Kruskal-Wallis test if the data were not normally distributed, followed by Dunn’s multiple comparisons post-test. Association between plasma proteins and clinical measurements and between mRNA-protein pairs were determined by Spearman correlation. All results were considered statistically significant at *p*-value <0.05, unless stated otherwise. Data visualization was conducted using R (v4.3.3) and Rstudio (v2023.09.0 + 463) with the ggplot2 R package (v3.5.0)^55^ and other extended R packages, including ComplexHeatmap (v2.18.0),^44^ ggthemes (v5.1.0).^45^

## Supplemental information

Document S1. Figures S1–S6 and Table S1

Data S1. Results of differentially expressed analysis in fibrosis-dependent analysis, related to Figures 2E, 5A, 6C, S2, S3B, S6A, and S6E

Data S2. Gene set enrichment analysis of differentially expressed genes, related to Figures 2C, 2D, S1D, S2, and S6

Data S3. Results of differentially expressed analysis in etiology-dependent analysis, related to Figure 2H

Data S4. Results of fuzzy clustering on plasma proteomics, related to Figures 4C and S4C

Data S5. Results of differential analysis on plasma proteomics, related to Figures 4D–4F, 5D, 6C, S4D–S4F, S5, and S6A

Data S6. Proteo-transcriptomics signatures, related to Figures 5B and 5C

Data S7. Protein importance in machine learning models for the prediction of advanced fibrosis and other model performance metrics, related to Figures 6C–6F

Document S2. Article plus supplemental information
