# Supplementary material for: Integrative proteo-transcriptomic characterization of advanced fibrosis in chronic liver disease across etiologies
Source: Cell Rep Med. 2025 Jan 30;6(2):101935. doi: 10.1016/j.xcrm.2025.101935 (PMC11866494; doi:10.1016/j.xcrm.2025.101935)
Supplement: Document S1. Figures S1–S6 and Table S1 [file mmc1.pdf]

Cell Reports Medicine, Volume *6*

Supplemental information

Integrative proteo-transcriptomic characterization of advanced fibrosis in chronic liver disease across etiologies

Hong Yang, Dila Atak, Meng Yuan, Mengzhen Li, Ozlem Altay, Elif Demirtas, Ibrahim Batuhan Peltek, Burge Ulukan, Buket Yigit, Tarik Sipahioglu, María Bueno Álvez, Lingqi Meng, Bayram Yüksel, Hasan Turkez, Hale Kirimlioglu, Burcu Saka, Cihan Yurdaydin, Murat Akyildiz, Murat Dayangac, Mathias Uhlen, Jan Boren, Cheng Zhang, Adil Mardinoglu, and Mujdat Zeybel

Supplemental information

Figures S1–S6 and Tables S1

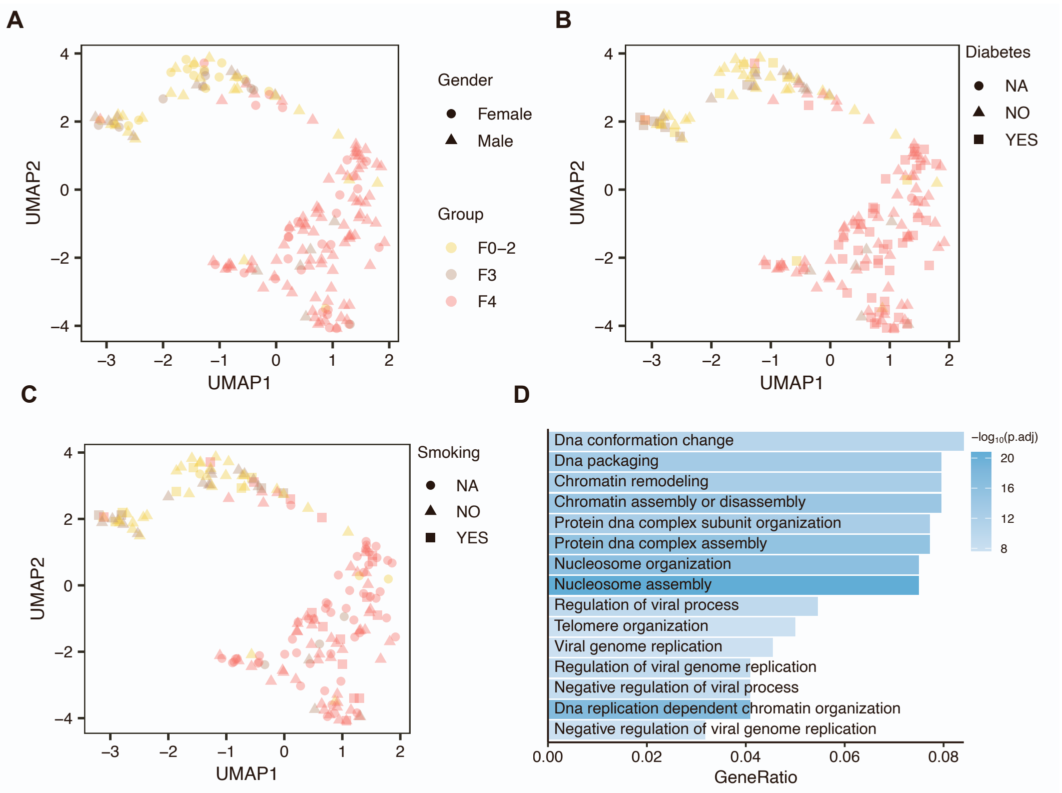

Fig. S1. **UMAP analysis on fibrosis stage-dependent liver transcriptome from all patients in the discovery cohort. Related to Fig. 2B&H**. (**A**) gender, (**B**) diabetes and (**B**) lifestyle. (**D**) The biological processes enriched (Benjamini and Hochberg false discovery rate adjusted P-value < 0.05) by genes that are differentially expressed in patients with MASLD compared to those with chronic viral hepatitis.

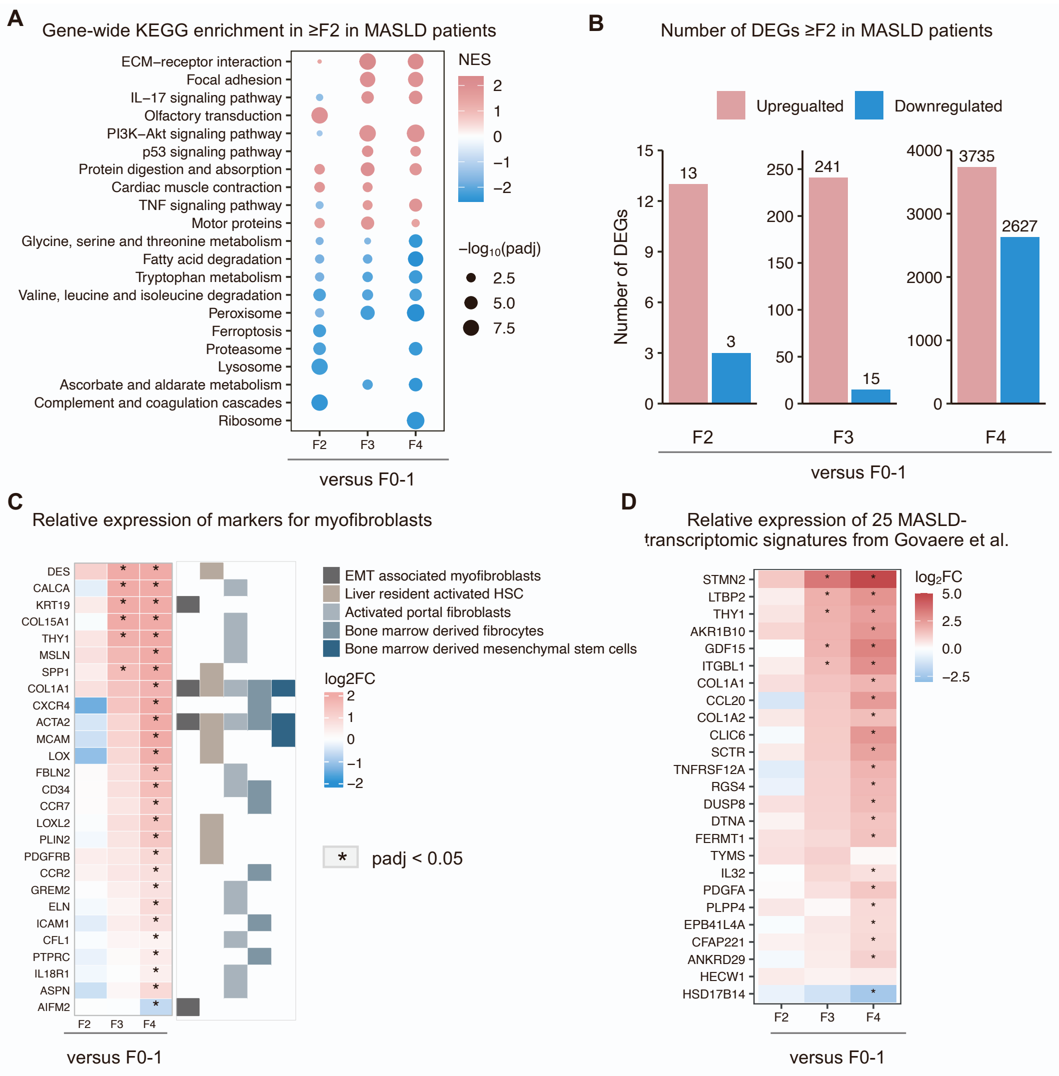

Fig. S2. **Transcriptomic changes associated with F≥2 in MASLD patients. Related to Fig. 2**. (**A**) Dot heatmap showing the top significantly regulated Kyoto Encyclopedia of Genes and Genomes (KEGG) pathways in samples with significant fibrosis or higher (F≥2) compared to those in F0/1 stages. (**B**) Bar plot showing the number of differentially expressed genes between samples with significant fibrosis (F≥2) and those in F0/1 stages. (**C**) Heatmap showing the relative expression (log2-Fold change) of fibrosis marker genes in hepatic tissues with significant fibrosis compared to those in F0/1/2 stages in patients with MASLD. (**D**) The relative expression of 25 MASLD-transcriptomic signatures from Govaere et al.[1] in hepatic tissues with significant fibrosis compared to those in F0/1/2 stages in this study. Abbreviation: EMT. epithelial-to-mesenchymal transition. MSC, mesenchymal stem cells. HSC, hepatic stellate cells.

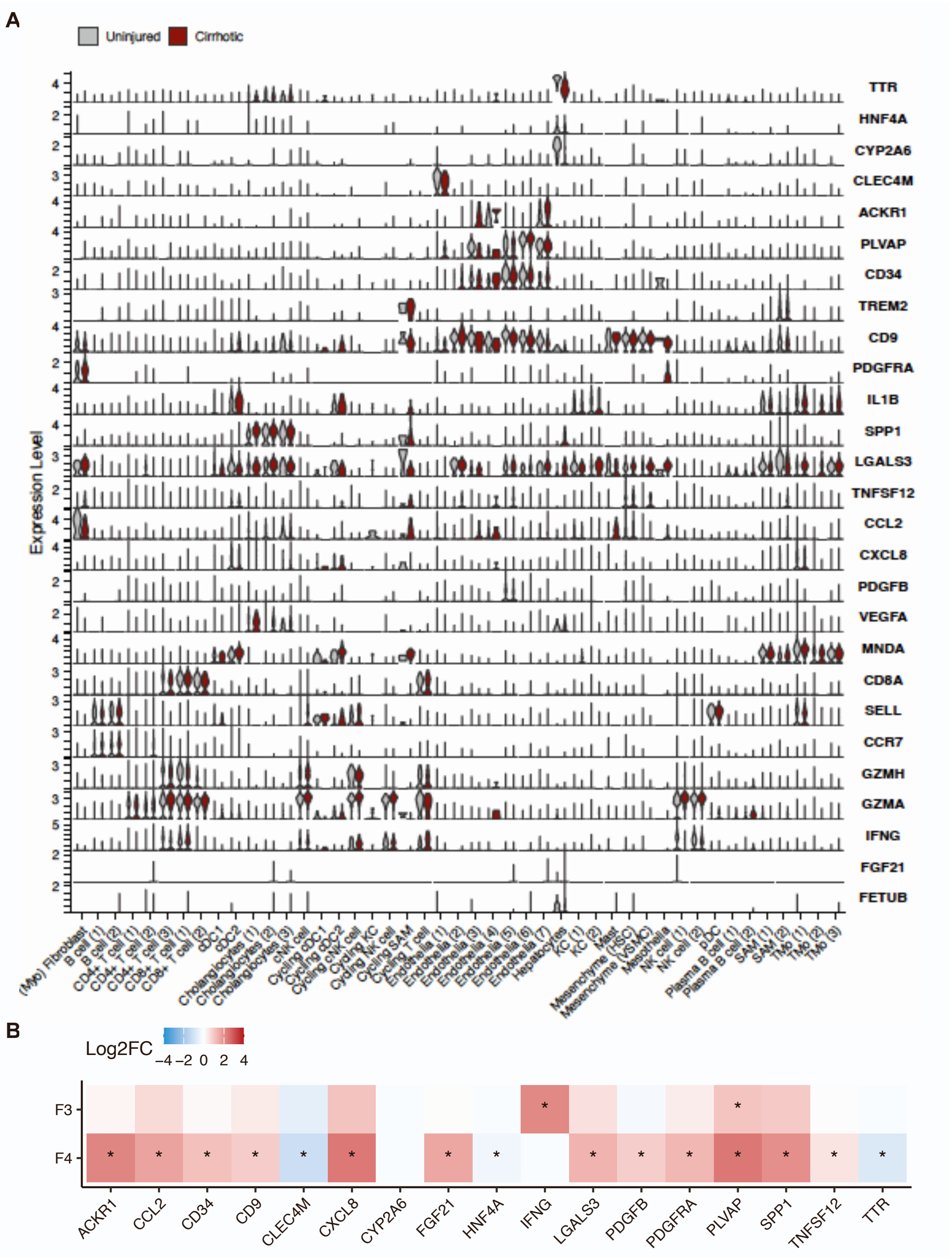

Fig. S3. **Gene expression of marker genes of specific cell type at both single-cell and bulk tissue level. Related to Fig. 3**. (**A**) Vlnplot showing the expression of genes across cell types analyzed in this study, related to Fig. 3. (**B**) The relative changes (Log2-Fold change) of marker genes in advanced stages of disease as compared to the F0-2 group.

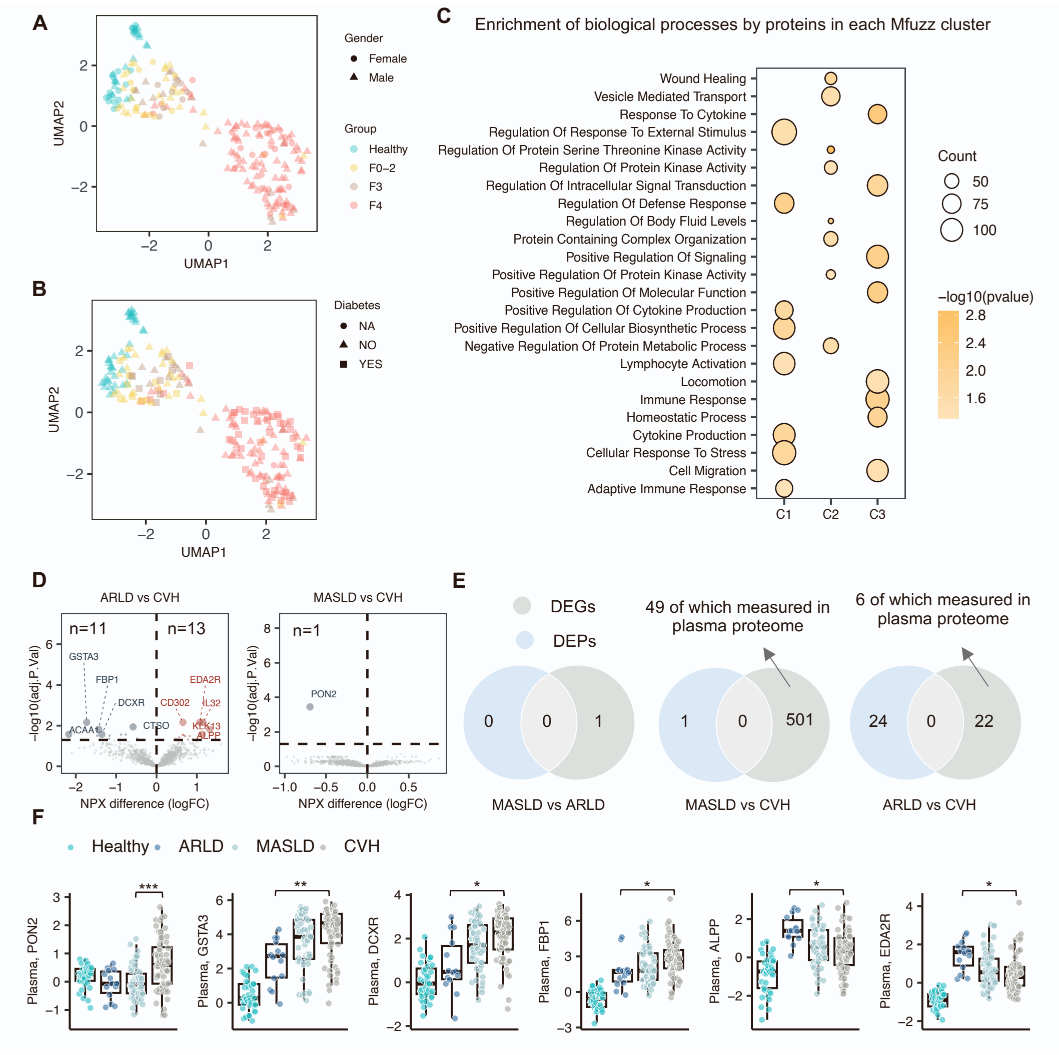

Fig. S4. **Fuzzy clustering of plasma proteome. Related to Fig. 5A&B**. (**A-B**) UMAP analysis on fibrosis stage-dependent plasma proteome from all patients in the discovery cohort based on gender and diabetes. (**C**) KEGG enrichment analysis on the genes in each fuzzy cluster. (**D**) Volcano plots showing the differentiated proteins in the groupwise comparisons. (**e**) Venn diagrams show the intersection between differentiated proteins and DEGs in the respective comparison among etiologies. (**F)** Boxplots showing the plasma levels of selected DEPs across the groups. *adjusted P-value < 0.05, **adjusted P-value < 0.01, ***adjusted P-value < 0.001 derived from DESeq2. This box plots represent the interquartile range (IQR), with the horizontal line indicating the median.

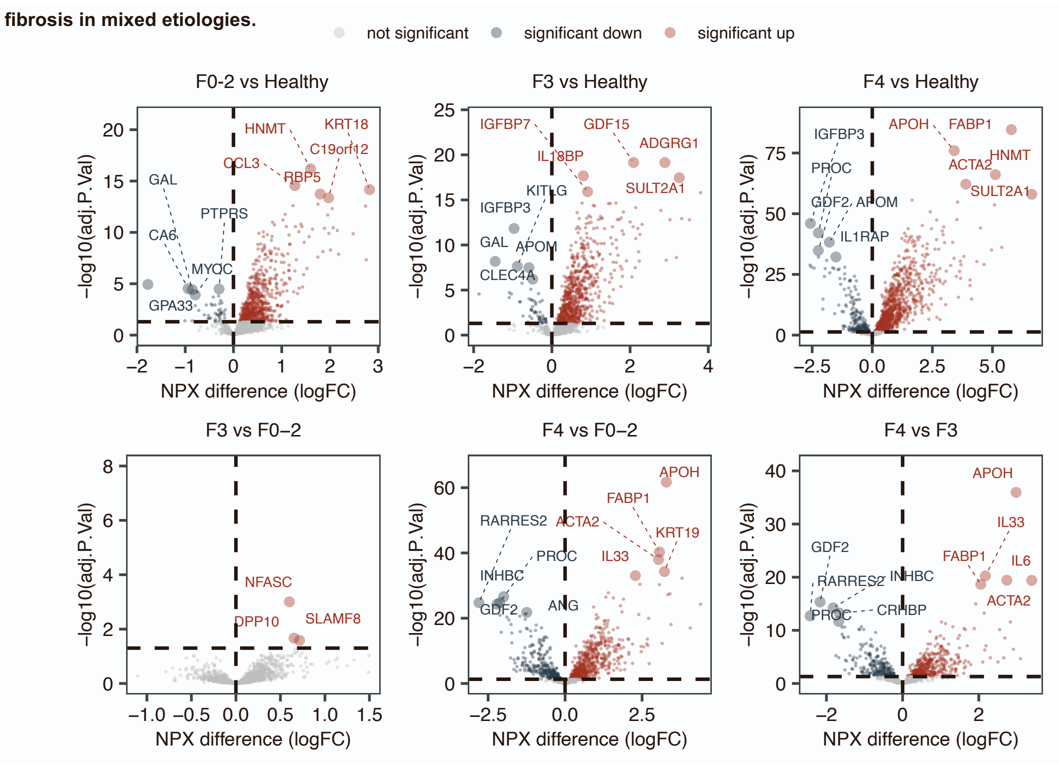

Fig. S5. **Volcano plots of different analyses of plasma proteomes. Related to Fig. 5**.

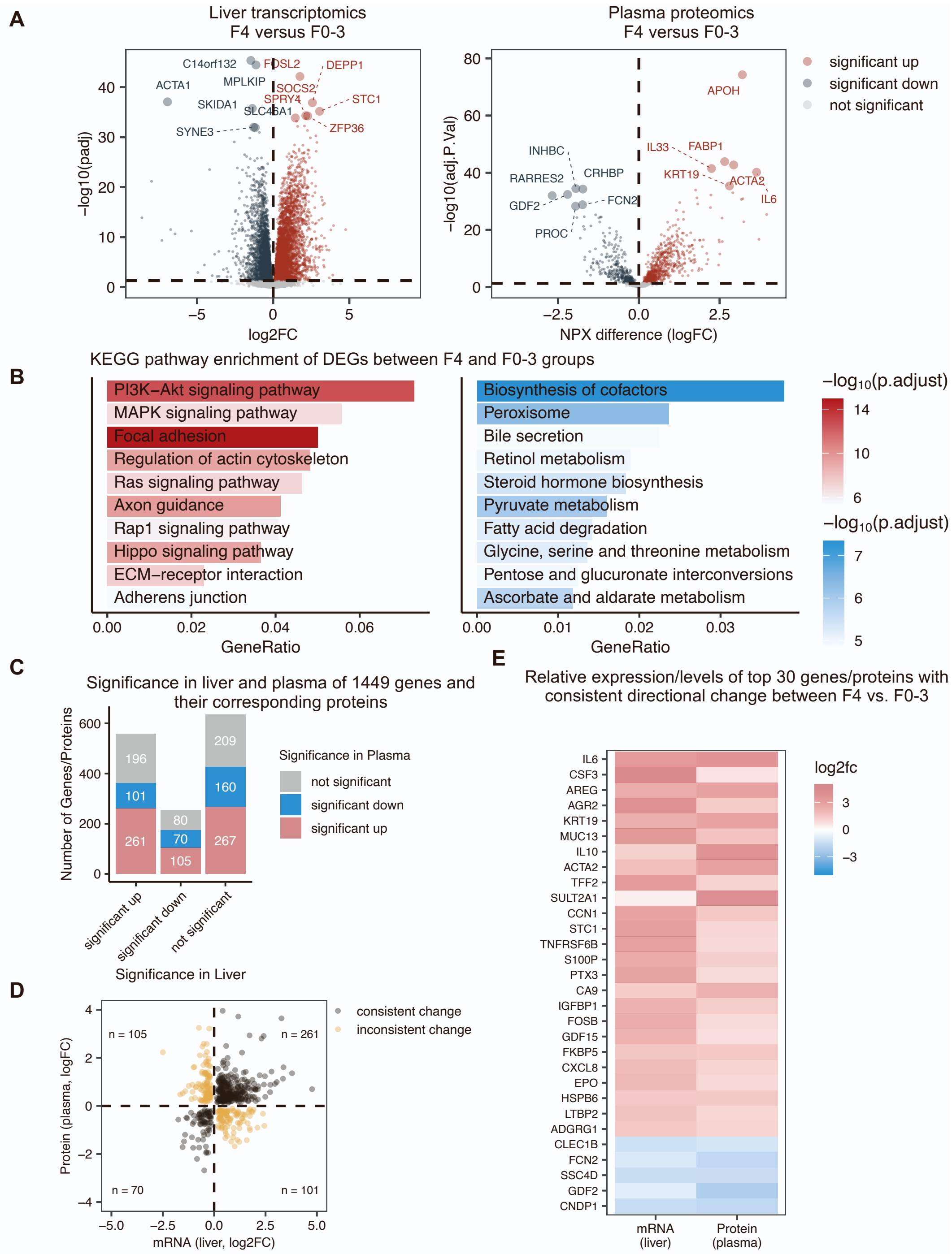

Fig. S6. Transcriptional and proteome changes between F4 versus F0-3. Related to **Fig. 2-4**. (**A**)Volcano plots showing differential gene expression (left panel) and protein levels (right panel) between F4 versus those in F0/1/2/3 groups. (**B**) Top 10 KEGG pathway enrichment of differentially expressed genes (DEGs) between F4 and F0/1/2/3 groups, with upregulated pathways shown in red and downregulated pathways in blue. (**C**) Bar plot comparing the significance of gene expression in liver and corresponding protein levels in plasma(upregulated, downregulated, or no change) between the two groups.(**D**) Scatter plot correlating differential gene expression in liver tissues with protein levels in plasma.(**E**) Heatmap showing the relative difference of the top-30 genes/proteins (log2FC) with consistent significant changes in both plasma and liver between the comparison groups.

Table S1. **Baseline participant characteristics of patients with viral hepatitis, MASLD, and HCC. Related to Table 1**

**Viral Hepatitis Characteristics**	**Discovery Cohort (n=58)**	**Validation Cohort (n=14)**
**Virus (n (%))**		
HBV	31 (53.4)	2 (14.3)
HCV	4 (6.8)	1 (7.1)
HBV+HCV	2 (3.4)	0
HBV+HDV	21 (26.4)	11 (78.6)
**MASLD Characteristics**	**Discovery Cohort (n=72)**	**Validation Cohort (n=53)**
**NAS Score (n (%))**		
< 3	37 (59.7)	10 (21.3)
⩾3	25 (40.3)	37 (78.7)
**HCC Characteristics**	**Discovery Cohort (n=34)**	**Validation Cohort (n=44)**
**AFP**	13 (4, 205)	20 (5, 482)
**BCLC Score (n (%))**		
0	5 (14.7)	7 (15.9)
A	12 (35.3)	13 (29.5)
B	16 (47.1)	17 (38.6)
C	1 (2.9)	5 (11.4)
D	0	2 (4.5)
**Baseline Etiology (n (%))**		
HBV	12 (35.3)	20 (45.5)
HCV	5 (14.7)	3 (6.8)
HBV+HCV	2 (5.9)	1 (2.3)
HBV+HDV	5 (14.7)	3 (6.8)
MASLD	10 (29.4)	13 (29.5)
ARLD	0	4 (9.1)

Data are presented as "frequencies (percentages)" for categorical variables and as "median (interquartile range)" for non-normally distributed variables. MASLD, metabolic dysfunction-associated steatotic liver disease.

Reference

1. Govaere, O., Cockell, S., Tiniakos, D., Queen, R., Younes, R., Vacca, M., Alexander, L., Ravaioli, F., Palmer, J., Petta, S., et al. (2020). Transcriptomic profiling across the nonalcoholic fatty liver disease spectrum reveals gene signatures for steatohepatitis and fibrosis. Sci Transl Med *12*. 10.1126/scitranslmed.aba4448.
